# Awareness of cervical cancer risk factors and preventive approaches, and perceived causes of cervical cancer among secondary school girls: a cross-sectional study in Northern Uganda

**DOI:** 10.1080/07853890.2024.2374860

**Published:** 2024-07-08

**Authors:** Stephen Oringtho, Amos Deogratius Mwaka, Christopher Garimoi Orach, Henry Wabinga

**Affiliations:** a Department of Community Health, Anaka General Hospital, Nwoya district, Gulu, Uganda; b Department of Public Health, School of Medicine, Uganda Christian University, Mukono, Uganda; c Department of Medicine, Faculty of Medicine, Gulu University, Gulu, Uganda; d Department of Community Health and Behavioural Sciences, School of Public Health, College of Health Sciences, Makerere University, Kampala, Uganda; e Department of Pathology, School of Biomedical Sciences, College of Health Sciences, Makerere University, Kampala, Uganda

**Keywords:** School girls, adolescence, awareness, cervical cancer, risk factors, human papillomavirus

## Abstract

**Background:**

The majority of women in low- and middle-income countries have low awareness of cervical cancer. This study sought to establish awareness of cervical cancer risk factors and preventive approaches, as well as sources of information and perceived causes of cervical cancer among secondary school girls in northern Uganda.

**Methods:**

This was a cross-sectional study conducted in rural northern Uganda. We collected data using an investigator administered pre-tested questionnaire. Analysis was done with STATA version 14.0. Multivariate analyses with logistic regressions models were used to determine magnitudes of association between independent and outcome variables. Odds ratios and accompanying 95% confidence intervals are reported. Statistical significance was considered if the two sided *p*-value <.05.

**Results:**

Most participants (97%; *n* = 624) had heard of cervical cancer before this study. The most common source of information about cervical cancer was friends (31.1%; *n* = 194). More than half of the participants (59%; *n* = 380) had heard about a vaccine that prevents cervical cancer, but only a third (33%; *n* = 124) had ever received a dose of the vaccine. The majority of participants (89%; *n* = 550) reported that cervical cancer could be prevented; however only half (52%; *n* = 290) knew that vaccination of girls aged 9–13 years could prevent cervical cancer. The majority of participants did not recognize the risk factors for cervical cancer; for example, only 15% (*n* = 98), 7% (*n* = 45), and 1.4% (*n* = 9) recognized early onset of sexual intercourse, infection by the human papillomavirus (HPV), and smoking respectively. On adjusting for age, students’ class, and religion, students in schools with school health programs were twice (aOR = 2.24: 95%CI; 1.24–4.06) more likely to know that cervical cancer is preventable.

**Conclusion:**

Secondary school girls need information on cervical cancer risk factors and approaches to prevention so that they may avoid exposures to the risk factors and promptly seek and undertake preventive approaches including HPV vaccinations.

## KEY MESSAGES

1. Educational interventions through school health programs are viable strategies to improve the knowledge of secondary school girls on cervical cancer risk factors and preventive approaches.
2. Peers/friends are key sources of information on cervical cancer to secondary school girls.
3. Peers/friends are a sustainable strategic resource, and therefore students could be trained to provide peer training on cervical cancer risk factors and preventive approaches to fellow secondary school girls.

## Background

Worldwide, there were an estimated 604,000 new cases and 342,000 deaths from cervical cancer in 2020, making cervical cancer the fourth most frequently diagnosed cancer among females. The burden of ­cervical cancer disproportionately affects women in low- and middle-income countries (LMICs). About 90% of all new cases and deaths from cervical cancer in 2020 occurred in the LMICs especially countries in South Eastern Asia and sub Saharan Africa (SSA) [1]. The highest incidence and mortality from invasive cervical cancer in women was registered in Southern & Eastern Africa countries [1,2]. In Uganda, cervical cancer was the most common cancer among women in 2020 with 6959 new cases and 4607 deaths from the disease [1,3]. In Uganda, cervical screening is opportunistic and conducted using visual inspection with acetic acid (VIA). Early cervical lesions are treated at the same visit with cryotherapy – the see and treat approach [4,5]. The cervical cancer screening guideline provided by the Uganda Ministry of Health recommends screening every 3 years for HIV negative women, and once every year for HIV positive women [4]. However, because of the opportunistic nature of the screening and multiple barriers to screening uptake, most women do not access and undertake screening [6]. Women who develop cervical cancer are often diagnosed with advanced stage disease [7,8]. Cervical cancer stage at diagnosis is one of the key prognostic factors that influence survival outcome [9–13]. The majority of women with cervical cancer in the LMICs are diagnosed at advanced stages of the disease, and experience low survival. Earlier studies in Uganda showed that more than 80% of women present late and are diagnosed with advanced stage cancer (Stage IV) and experience low survival rates [14–16]. Low mortality in high-income countries is attributed to awareness and diagnosis of cervical cancer in early stages and prompt institution of quality cancer specific treatments of invasive cervical cancer [17,18].

Awareness of the risk factors and symptoms of cancer has been associated with timelier presentations and earlier stage of the disease at diagnosis including pre-malignant lesions [19–22]. However, awareness of cervical cancer risk factors and symptoms is low among both secondary and high school girls in high incidence areas. A study that involved 400 students from two schools in Nigeria showed that awareness of cervical cancer and human papilloma virus (HPV) was low at 36% (*n* = 142), and 32% (*n* = 128) respectively; none of the participants reported having had any dose of the HPV vaccine [23]. Similarly, low awareness of cervical cancer and preventive measures was associated with low uptake of screening in Nigeria [24]. The low awareness of cervical cancer risk factors and symptoms among young girls may contribute to their vulnerability and exposures to cervical cancer risks including early age at sexual debut and multiple pregnancies. A study that analyzed data on young women aged 10–24 years from 20 districts in Uganda showed that 13% (*n* = 581) had their sexual debut at ages less than 15 years, and 51% (*n* = 2265) at ages 15-17 years. For girls at school, 8% (*n* = 114) had a sexual debut before the age 15 years while 49% (*n* = 721) had a sexual debut between the age 15 – 17 years [25]. The prevalence of condom use during sex was low at just 20% (*n* = 728) for the most recent sexual experience [25]. An analysis of the Uganda Demographic and Health Survey (UDHS) data from 1988 to 2016 showed that the prevalence of child birth among girls aged 15–19 years remained high at about 25% [26]. Young women aged 15–19 years in rural areas were more likely to start child bearing earlier than in urban centers; 26.7% (*n* = 3230) versus 18.8% (*n* = 1034) [27]. In the Acholi sub region, which is part of northern Uganda, 24% (*n* = 246) of female aged 15–19 years were reported to have started child bearing compared to 17% (*n* = 200) in Kampala, and 15% (*n* = 162) in Kigezi in south western Uganda (UBOS and ICF, 2017). In Uganda, the teenage pregnancy rate has remained high at 26%, 25% and 26% for the years 2006, 2011 and 2016 respectively [28]. Teenage pregnancy was more likely among women aged 15 – 19 years who experienced early sexual debut compared to those who did not experience early sexual debut; AOR = 21.09 (95%CI: 13.18–33.74) for 2006; AOR = 18.61(95%CI:11.44–30.27) for 2011; and AOR = 22.84 (95%CI:16.45–31.70) for 2016 Demographic Health Surveys [28]. Awareness of young women about cervical cancer risks and prevention approaches including delay of sexual debut and uptake of HPV vaccination and screening potentially helps to mitigate the burden of invasive cervical cancer in the LMICs. This study therefore sought to describe the awareness of cervical cancer risk factors and preventive approaches, and sources of information and perceived causes of cervical cancer among secondary school girls in northern Uganda so as to inform interventions to increase uptake of preventive measures including HPV vaccination.

## Methods

### Study design and setting

This was a cross-sectional study. The study was conducted in all the eleven secondary schools in Nwoya district, northern Uganda. In the 2014 National Census, Nwoya district had a total population of 133,509 inhabitants. The population of young people aged 10–19 years was 34, 296 (16,911 were female) [29]. The projected population in 2019 show a total population of 214,200 (female = 107,700); and young people aged 10–19 years of 55,040 (female = 26,970). The projected population of young people aged 12–19 years in 2019, were 42,410 (female = 20,940) [30]. Nwoya district, like the other districts in northern Uganda, experienced a violent civil conflict between the government of Uganda and the Lord’s Resistant Army (LRA) rebels that lasted for two decades (1986–2006). The conflict forced more than 90% of the people of northern Uganda into congested Internally Displaced Persons (IDP) Camps where they stayed until 2006 when calm was re-established [31,32]. This study setting was chosen because there was a need to gain insights into the knowledge status of the young people who may have exposed themselves to violent sexual encounters and hence high possibility of acquiring the Human papilloma virus (HPV) [33–35] that is causally associated with cervical cancer, and the human immunodeficiency virus (HIV) that predisposes to development and manifestations of invasive cervical cancer [36–38]. The two decades insurgency did not only affect healthcare and education services delivery but also led to loss of morals, increased transactional sex, and cultural degenerations, with consequent high rates of both HPV and HIV infections [39–42].

### Study participants and sample size

The study involved adolescent secondary school girls, aged 12–19 years, in their ordinary level (O-level) education, i.e. senior one to senior four. The first author (SO) visited the office of the district education officer (DEO) of Nwoya district. He obtained records on both public and private secondary schools in the district. There were eleven secondary schools in the district during the study period. Three of the eleven secondary schools comprised of both Ordinary (O) and Advance (A) levels. The total number of adolescent girls aged 12–19 years in O-level, in all eleven secondary schools was 719. Eligible participants included all adolescent girls aged 12–19 years, enrolled in senior one to senior four in all the secondary schools in Nwoya district. Only adolescent girls who provided informed consent and whose parents or guidance provided assents were recruited into the study. We excluded adolescent girls registered in schools but who were absent in the schools during the study period; reasons for absence included sickness, and non-payment of schools fees.

The sample size was calculated based on the prevalence estimate for awareness of cervical cancer risk factors in the population of adolescent secondary school girls in the ordinary level. Since there was no similar study results in a similar setting, a prevalence of 50% awareness was assumed in the formula for sample size estimation for cross-sectional studies by Kish Leslie [43]. The estimated sample size was 384; to allow for variations in participants’ responses due to cluster effects between schools (e.g. public versus private, and locations by sub-counties) which potentially introduces selection bias, we increased the sample size by introducing a design effect of two. The computed sample size was therefore 768 participants.

### Participant recruitment and sampling procedure

We sampled participants from the Ordinary (O) level so that the data informs interventions targeting a specific identifiable category of girls who may still have limited exposure to cervical cancer risk factors including sexual debut and multiple sexual partnership. The Advance (A) level students were older and more likely to have got more exposure, yet only three (3) schools had Advance (A) level. Participants were recruited by consecutive sampling technique. All the eligible students were approached and enrolled into the study.

### Data collection

Data collection was conducted during July and August 2019. Two of the research members (SO and ADM) trained eight female research assistants (RA) on aspects of cervical cancer risk factors and symptoms, the study purpose, objectives, recruitment, and consenting procedures. The research assistants (RAs) were university graduates who have vast field experiences in both quantitative and qualitative data collection. They had participated in the African women awareness of cancer (AWACAN) project to develop and validate a breast and cervical cancer awareness measurement tool for sub Saharan Africa [44]. The RAs participated in the pre-testing of the study tool in a school in Gulu Municipality after the training. The RAs worked in pairs both during the pre-test and study data collection. The study tool was refined based on the data collected during the pre-test. The tool has sections including on demographic characteristics, school characteristics, community factors, and questions on sources of information on cervical cancer as well as cervical cancer risk factors and perceived causes (Supplementary material 1). Data were collected from all eleven secondary schools in the district. The eight RAs went to one school at a time, and worked in pairs to collect data till all the selected schools were covered. SO supervised the research assistants during data collection; he visited each of the schools on the days of data collection.

### Data management and analysis

The research team, under the supervision of SO reviewed all the questionnaires for completeness before leaving the schools at the end of each data collection day. Data entry was done by an experienced data clerk using Epidata 3.1. The laptop into which data was entered was password protected. SO reviewed the dataset as entry was ongoing, and ensured completeness, accuracy and consistency between soft data and the paper questionnaire. The biostatistician reviewed 10% of randomly selected questionnaires to ensure data quality before data analysis. There were no significant inconsistencies detected in data capture. Data were exported to STATA I/C version 14.0 for analysis. The demographic characteristics of participants were summarized using means with respective standard deviations, medians with interquartile ranges, and proportions. At bivariate analysis, we used Chi square tests to determine associations between the socio-demographic characteristics of participants and the outcome variables. At multivariate analyses, we used unconditional logistic regression models to determine magnitudes of associations between binary outcome variables (Yes = 1, and No = 0) and the independent variables. Predictor variables were included into the multivariable model based on pre-determined selection criteria depending on biological plausibility and their use in similar previous studies. The measure of effect sizes was the odds ratios and accompanying 95% confidence intervals. Two sided p-values < 0.05 were considered cut off for determining statistical significance. Two risk factors, sexual debut and multiple sexual partnership, were purposefully selected for analysis of predictor variables. These are risk factors that are critical for interventions on cervical cancer prevention and control at this age group. In Uganda HPV vaccination of girls aged at least 10 years is recommended mainly in primary schools, and therefore evaluation and promotion of its uptake requires participants from primary schools [45,46]. Evaluation and interventions on smoking and multi-parity are relevant to females at older ages, perhaps advanced levels, and higher institutions of learning.

### Ethical considerations

The study was approved by the Uganda Christian University Research Ethics Committee (UCU REC), reference number 02-05-600-000124. We obtained permission to access schools from the District Education Officer and the Head teachers of the respective schools. A week to the onset of data collection, SO visited the schools and held meetings with the class teachers. They discussed consent and assent procedures. The assent forms were distributed to the class teachers who handed them to the day scholar students to take to their respective parents/guardians. Students were also asked to provide the mobile telephone numbers of their parents or guardians so that the research team or class teachers could reach out to them to explain the purpose, objectives and procedures of the study, and their need to sign, thumb print or provide expressed verbal permissions to the class teachers to sign the assent forms on their behalf. The class teachers who are known to the parents called the parents and explained to them the need for the study and especially that there would not be any invasive procedures nor any samples taken from the students. On the days of data collection, the research assistants again explained the purpose and procedure of the study to all participants and obtained individual written informed consents/assents for minors before conducting interviews. Participants were informed of their freedom to decline participation if they chose to and or join the study and withdraw at any point without fear of retribution from the study team or the school administration. The participants were told that personal information about them would be kept confidential to minimize the risk of exposure to unauthorized persons. They were also told that the findings from the study can inform important policies on cervical cancer prevention and control especially among secondary school girls. In particular, participants were assured that data would be anonymized and codes used during analysis, and that their names or any potential identifier information will not be included in the dissertation and publication of findings.

## Results

### Study participants

About half of the schools (55%; 6/11) were private own, while the majority of participants were aged 16–19 years (78%; 499/644), median age = 16 years (interquartile range; 16–17). The majority of participants (78%; 505/644) lived at or more than 5 kilometers from the nearest health facility (Table 1).

**Table 1.** Socio-demographic Characteristics of participants.

Characteristics	Frequency	Percentage
**School ownership; *N* = 11**		
Private schools	6	54.5
Public schools	5	45.5
**Students enrollment in the study; *N* = 644**		
Private schools	308	47.8
Public schools	336	52.2
**Age group (years); *N* = 644**		
12 – 15	145	22.5
16 – 19	499	77.5
Median age (Interquartile Range)	16 (16–17)	
**Class; *N* = 644**		
Senior 1	202	31.4
Senior 2	185	28.7
Senior 3	136	21.1
Senior 4	121	18.8
**Religion; *N* = 644**		
Roman Catholics	377	58.5
Anglicans/Protestants	166	25.8
Pentecostals	93	14.4
Muslims	7	1.1
Others	1	0.2
**Level of education of fathers; *N* = 607**		
No formal education	19	3.1
Primary education	188	31.0
Secondary education	273	45.0
Tertiary education	56	9.2
Not aware	71	11.7
**Level of education of mothers; *N* = 636**		
No formal education	71	11.2
Primary education	385	60.5
Secondary education	130	20.4
Tertiary education	13	2.1
Not aware	37	5.8
**Distance to nearest health facility (kilometers - km); *N* = 644**		
< 5 km	139	21.6
≥ 5 km	505	78.4

### Awareness, sources of information, and perceptions about cervical cancer

Most participants (97%; *n* = 624) had heard of cervical cancer before this study. The most common source of information about cervical cancer was friends (31.1%; *n* = 194). Although more than half of participants (59%; *n* = 380) had heard about a vaccine that prevents cervical cancer, only a few (33%; *n* = 124) had ever received a dose of the vaccine (Table 2).

**Table 2.** Cervical cancer awareness, sources of information, prevention and self-perceived risk.

Issue assessed	Frequency	Percentage
**A: Awareness of cervical cancer**		
**Ever heard about cervical cancer; *N* = 643**		
Yes	624	97.1
No	19	2.9
**B: Sources of knowledge on cervical cancer**		
**Main source of information about cervical cancer; *N* = 624**		
Friends	194	31.1
Health workers	143	22.9
School	99	15.7
Radio	66	10.6
News papers	35	5.6
Television	26	4.2
Family members	22	3.5
Neighbors	16	2.6
Churches/ Mosques	6	1.1
Others	17	2.7
**C: Cervical cancer prevention awareness and practices**		
**Ever heard of vaccine against cervical cancer, *N* = 644**		
Yes	380	59.0
No	264	41.0
**Ever been vaccinated against cervical cancer, *N* = 380**		
Yes	124	32.6
No	256	67.4
**Number of times vaccinated; *N* = 124**		
Once	57	46.0
Twice	52	41.9
Thrice	15	12.1
**Cervical cancer can be prevented, *N* = 620**		
Yes	550	88.7
No	56	9.0
Do not know	14	2.3
**Cervical cancer can be prevented through:**		
**(i) Vaccination of young girls before sex; *N* = 556**		
Yes	290	52.2
No	266	47.8
**(ii) Frequent vaginal examination at a health facility; *N* = 555**		
Yes	157	28.3
No	398	71.7
**D: Self-perceived risk of cervical cancer**		
**Perceives self to be at risk of cervical cancer; *N* = 639**		
Yes	150	23.5
No	420	65.7
Do not know	69	10.8

Only a few (24%; *n* = 150) participants perceived themselves to be at risk of developing cervical cancer in their lifetime. The majority of participants (89%; *n* = 550) reported that cervical cancer could be prevented; however, only about half (52%; *n* = 290) knew that vaccination of young girls against the HPV virus could prevent cervical cancer. Less than half were aware that invasive cervical cancer could be prevented through repeated Pap smear tests (28%; *n* = 157) (Table 2).

### Awareness of cervical cancer risk factors and perceived causes of cervical cancer

The majority of participants could not recognize the risk factors for cervical cancer. For example, only 15% (*n* = 98), 7% (*n* = 45), and 1.4% (*n* = 9) recognized the early onset of sexual intercourse, infection by the human papillomavirus (HPV) and smoking respectively. The most commonly perceived causes of cervical cancer were recognized as not causal of the disease. For example, 89% (*n* = 569), 74% (*n* = 475), 73% (*n* = 466) and 66% (*n* = 421) reported that cervical cancer is not caused by having sex with a polygamous man, having rough sexual intercourse, not cleaning the genital, and having sexual intercourse during menstruation respectively (Table 3).

**Table 3.** Recognition of cervical cancer risk factors and perceived causes of cervical cancer.

Characteristics	Frequency	Percentage
**A: Epidemiologic risk factors for cervical cancer**		
**Early onset of sexual activity before age 11 years; *N* = 642**		
True	98	15.3
False	468	72.9
Not sure	76	11.8
**Getting infected with HPV; *N* = 640**		
True	45	7.0
False	509	79.5
Not sure	86	13.5
**Multiple sexual partnership; *N* = 642**		
True	47	7.3
False	538	83.8
Not sure	57	8.9
**Smoking cigarettes/tobacco; *N* = 638**		
True	9	1.4
False	178	27.9
Not sure	451	70.7
**Bearing many children (> 5 children); *N* = 633**		
True	11	1.7
False	216	34.1
Not sure	406	64.1
**B: Perceived causes of cervical cancer**		
**Being poor; *N* = 642**		
True	1	0.2
False	154	24.0
Not sure	487	75.8
**Having sex with a polygamous man; *N* = 641**		
True	9	1.4
False	569	88.8
Not sure	63	9.8
**Rough sexual intercourse; *N* = 639**		
True	3	0.5
False	475	74.3
Not sure	161	25.2
**Having sex with a man who does not believe in God; *N* = 641**		
True	170	26.5
False	470	73.3
Not sure	1	0.2
**Cervical cancer is contagious; *N* = 639**		
True	2	0.3
False	53	8.3
Not sure	584	91.4
**It is inherited; *N* = 640**		
True	3	0.5
False	58	9.0
Not sure	579	90.5
**Not cleaning a woman’s genital after sexual intercourse; *N* = 639**		
True	13	2.0
False	466	72.9
Not sure	160	25.1
**Having sex during menstruation; *N* = 640**		
True	6	0.9
False	421	65.8
Not sure	213	33.3

### Factors associated with sexual debut and multiple sexual partnerships

There were no statistically significant association between age, student’s class, religion and whether or not a student is in a private or public school with cervical cancer risk factors including early onset of sexual intercourse (Table 4).

**Table 4.** Socio-demographic factors and early sexual debut as the cause of cervical cancer.

	Cervical cancer is caused by early sexual debut		Crude Odds ratio (95%CI)	*Adjusted Odds ratio (95%CI)
Characteristics	Yes N (%)	No N (%)		
**Age group (years); *N* = 624**				
12–15	18 (19.2)	119 (22.5)	1.00	1.00
16–19	76 (80.8)	411 (77.5)	1.22 (0.70–2.12)	1.08 (0.55–2.09)
**Class; *N* = 624**				
Senior 1	27 (28.7)	164 (30.9)	1.00	1.00
Senior 2	19 (20.2)	162 (30.6)	0.71 (0.38–1.33)	0.69 (0.35–1.35)
Senior 3	26 (27.7)	106 (20.0)	1.49 (0.82–2.69)	1.38 (0.70–2.72)
Senior 4	22 (23.4)	98 (18.5)	1.36 (0.74–2.52)	1.30 (0.64–2.64)
**Religion; *N* = 624**				
Roman Catholics	55 (58.5)	311 (58.7)	1.00	1.00
Anglicans/Protestants	24 (25.5)	136 (25.7)	1.00 (0.59–1.68)	1.01 (0.60–1.71)
Pentecostals	14 (14.9)	76 (14.3)	1.04 (0.55–1.97)	1.10 (0.58–2.10)
Muslims	0 (0.0)	7 (1.3)	NA	–
Others	1 (1.1)	0 (0.0)	NA	–
**Category of schools; *N* = 624**				
Private schools	38 (40.4)	262 (49.4)	1.00	1.00
Public schools	56 (59.6)	268 (50.6)	1.44 (0.92–2.25)	1.37 (0.87–2.16)
**Presence of school dispensary; *N* = 624**				
No	77 (81.9)	424 (80.0)	1.00	1.00
Yes	17 (18.1)	106 (20.0)	0.88 (0.50–1.56)	0.84 (0.47–1.50)
**Presence of school health program; *N* = 618**				
No	48 (51.6)	269 (51.2)	1.00	1.00
Yes	45 (48.4)	256 (48.8)	0.99 (0.63–1.53)	0.96 (0.61–1.50)
**Perceived risk to cervical cancer; *N* = 621**				
Yes, I am at risk	19 (20.2)	130 (24.7)	1.00	1.00
No, I am not at risk	68 (72.3)	340 (64.5)	1.37 (0.79–2.37)	1.58 (0.89–2.79)
Do not know	7 (7.5)	57 (10.8)	0.84 (0.33–2.11)	0.97 (0.38-–2.49)
**Distance to nearest health facility (kilo meters – km); *N* = 624**				
≥5 km	77 (81.9)	413 (77.9)	1.00	1.00
< 5 km	17 (18.1)	117 (22.1)	0.78 (0.44–1.37)	0.78 (0.44–1.39)
**Sexual experience; *N* = 613**				
Ever had sex	17 (18.1)	82 (15.5)	1.00	1.00
Never had sex	77 (81.9)	442 (83.5)	0.84 (0.47–1.49)	0.96 (0.52–1.78)
Prefer not to mention	0 (0.0)	5 (1.0)	NA	–

* Adjusted for all factors in the table; Bold face = Factors statistically significant; NA: Not Applicable (numbers too few).

Participants who were in higher classes, i.e. senior two to four were two to six times more likely to know that multiple male sexual partnership is a risk factor for cervical cancer, after adjusting for all the variables as shown in Table 5 including age, religion, and presence of school health programs.

**Table 5.** Socio-demographic factors and multiple sexual partners as the cause of cervical cancer.

	Cervical cancer is caused by having sexual intercourse with multiple sexual partners		Crude Odds ratio (95%CI)	*Adjusted Odds ratio (95%CI)
Characteristics	Yes N (%)	No N (%)		
**Age group (years); *N* = 624**				
12–15	14 (29.79)	123 (21.32)	1.00	1.00
16–19	33 (70.21)	454 (78.68)	0.64 (0.33–1.23)	0.25 (0.11–0.60)
**Class; *N* = 624**				
Senior 1	8 (17.02)	183 (31.72)	1.00	1.00
Senior 2	15 (31.91)	166 (28.77)	2.07 (0.85–5.00)	**3.70 (1.39–9.84)**
Senior 3	12 (25.53)	120 (20.80)	2.29 (0.91–5.76)	**5.43 (1.79–16.51)**
Senior 4	12 (25.53)	108 (18.72)	2.54 (1.01–6.41)	**6.27 (1.99–19.74)**
**Religion; *N* = 624**				
Roman Catholics	26 (55.32)	340 (58.93)	1.00	1.00
Anglicans/Protestants	12 (25.53)	148 (25.65)	1.06 (0.52–2.16)	0.98 (0.47–2.02)
Pentecostals	9 (19.15)	81 (14.04)	1.45 (0.66–3.22)	1.38 (0.61–3.12)
Muslims	0 (0.00)	7 (1.21)	NA	–
Others	0 (0.00)	1 (0.17)	NA	–
**Category of school; *N* = 624**				
Private schools	20 (42.55)	280 (48.53)	1.00	1.00
Public schools	27 (57.45)	297 (51.47)	1.27 (0.70–2.32)	1.21 (0.65–2.24)
**Presence of school dispensary; *N* = 624**				
No	40 (85.11)	461 (79.90)	1.00	1.00
Yes	7 (14.89)	116 (20.10)	0.70 (0.30–1.59)	0.77 (0.33–1.79)
**Presence of school health program; *N* = 618**				
No	27 (57.45)	290 (50.79)	1.00	1.00
Yes	20 (42.55)	281 (49.21)	0.76 (0.42–1.39)	0.73 (0.39–1.34)
**Perceived risk of cervical cancer; *N* = 621**				
Yes, I am at risk	11 (23.40)	138 (24.04)	1.00	1.00
No, I am not at risk	27 (57.45)	381 (66.38)	0.89 (0.43–1.84)	0.91 (0.43–1.93)
Do not know	9 (19.15)	55 (9.58)	2.05 (0.81–5.23)	2.39 (0.92–6.24)
**Number of boyfriends; *N* = 351**				
One only	26 (83.87)	238 (74.38)	1.00	1.00
More than one	5 (16.13)	82 (25.62)	0.56 (0.21–1.50)	0.55 (0.20–1.50)
**Sexual experience; *N* = 623**				
Ever had sex	10 (21.28)	89 (15.45)	1.00	1.00
Never had sex	37 (78.72)	482 (83.68)	0.68 (0.33–1.42)	0.69 (0.31–1.54)
Prefer not to mention	0 (0.00)	5 (0.87)	NA	
**Distance to nearest health facility (km), *N* = 624**				
≥5 km	39 (82.98)	451 (78.16)	1.00	1.00
< 5 KM	8 (17.02)	126 (21.84)	0.73 (0.33–1.61)	0.78 (0.35–1.74)

* Adjusted for all factors in the table; Bold face = Factors statistically significant, NA: Not applicable (low cell counts).

### Factors associated with perception that cervical cancer is preventable

There was no association between the perception that cervical cancer is preventable with age, students’ class, religion, and being in public or private school. However, after adjusting for age, students’ class and religion, students in schools with school health programs were twice as likely to know that cervical cancer is preventable (Table 6).

**Table 6.** Socio-demographic factors and knowledge that cervical cancer is preventable.

Characteristics	Cervical cancer is preventable		Crude Odds Ratio (95%CI)	*Adjusted Odds Ratio (95%CI)
	Yes *N* (%)	No *N* (%)		
**Age group (years); *N* = 606**				
12–15	118 (21.5)	12 (21.4)	1.00	1.00
16–19	432 (78.6)	44 (78.6)	1.00 (0.51–1.95)	0.67 (0.31–1.46)
**Class; *N* = 606**				
Senior 1	163 (29.6)	23 (41.1)	1.00	1.00
Senior 2	163 (29.6)	13 (23.2)	1.77 (0.87–3.61)	2.06 (0.96–4.44)
Senior 3	118 (21.5)	9 (16.1)	1.85 (0.83–4.14)	2.31 (0.96–5.56)
Senior 4	106 (19.3)	11 (19.6)	1.36 (0.64–2.90)	1.66 (0.72–3.85)
**Religion; *N* = 606**				
Roman Catholics	324 (58.9)	33 (58.9)	1.00	1.00
Anglicans/Protestants	140 (25.5)	17 (30.4)	0.84 (0.45–1.56)	0.82 (0.44–1.53)
Pentecostals	79 (14.4)	6 (10.7)	1.34 (0.54–3.31)	1.38 (0.56–3.43)
Muslims	6 (1.1)	0 (0.0)	NA	
Others	1 (0.2)	0 (0.0)	NA	
**Category of school; *N* = 606**				
Private schools	259 (47.1)	29 (51.8)	1.00	1.00
Public schools	291 (52.9)	27 (48.2)	1.21 (0.70–2.09)	1.22 (0.70–2.13)
**Distance to nearest health facility (kilometers-km); *N* = 606**				
≥5 km	433 (78.7)	43 (76.8)	1.00	1.00
< 5 km	117 (21.3)	13 (23.2)	0.89 (0.47–1.72)	0.89 (0.46–1.73)
**School has dispensary; *N* = 606**				
No	439 (79.8)	48 (85.7)	1.00	1.00
Yes	111 (20.2)	8 (14.3)	1.52 (0.70–3.30)	1.59 (0.73–3.49)
**School has school health program; *N* = 600**				
No	266 (48.9)	38 (67.9)	1.00	1.00
Yes	278 (51.1)	18 (32.1)	**2.21 (1.23–3.96)**	**2.24 (1.24–4.06)**

* Adjusted for all factors on table; Bold = Factors statistically significant, NA: Not applicable (low cell counts).

## Discussions

Most of the secondary school girls aged 12–19 years in the public and private schools in Nwoya district, northern Uganda had heard about cervical cancer before this study. Friends/peers were the most common source of information about cervical cancer. The majority of participants were aware that cervical cancer is preventable. Participants from schools with health programs were twice as likely to know that cervical cancer is preventable. However, most participants were not aware of how cervical cancer can be prevented. Just half of the participants knew that vaccination of girls before sexual debut could prevent cervical cancer, while less than half knew that the Pap smear test is a preventive approach for invasive cervical cancer. About one in 10 participants who had ever heard of the HPV vaccine (*N* = 380) had ever received a dose of the vaccine. Only one in five participants perceived themselves to be at risk of developing cervical cancer.

There was low awareness about the HPV vaccine and this may undermine the uptake of the vaccine. Indeed, less than half of the girls in this study had received even a single dose of the HPV vaccine. Our results are similar to that of a study in the neighboring Lira district, that included 460 adolescents aged 12–17 years (94%; 432/460 were school going girls). In that study, only half of the participants had received any dose of the HPV vaccine, while 18% had received one dose, and 15% received two doses [47]. In a secondary school in central Uganda, only 9.2% of 380 students aged 13 – 19 years had ever had HPV vaccination [48]. Similarly, another study in Uganda that included female aged 9 – 15 years recruited from 56 villages showed that only 49% (200/407) had initiated the vaccination [49]. In Nigeria, a study among 321 female secondary students showed that only 23% and 22% had heard of Pap smear and HPV vaccinations respectively [50]. Another study (*N* = 2530) in Nigeria that involved both girls (44%) and boys in secondary schools showed that knowledge of HPV (23%) and HPV vaccines (18.3%) were low [51]. Similarly low awareness (41.5%; *n* = 257) and poor attitudes towards the HPV vaccine was reported in a study in Ethiopia among 620 students aged 15 – 24 years [52]. However, a study in Ethiopia among 366 high school students aged 16 – 20 years revealed majority (70%, *n* = 257) had heard of the HPV vaccine and 76% (*n* = 277) were willing to take the vaccine. Up to 65% (*n* = 238) of the participants in that study knew that HPV is sexually transmitted [53]. Knowledge of HPV and vaccine seems to improve as students get older and advance with their education. The younger students in lower classes therefore need urgent attention to protect them from exposure and increased vulnerability to invasive cervical cancer.

Awareness of the epidemiologic risk factors for cervical cancer including early onset of penetrative vaginal sexual intercourse, infections with HPV, engagement with multiple male sexual partners, and smoking cigarettes was extremely low among the secondary school girls in this study. Yet, avoiding exposure to the risk factors of cervical cancer is important for young girls to reduce their chances of developing invasive cervical cancer. In a study in central Uganda (*N* = 380 students), involvement in penetrative vaginal sexual intercourse at the low age of 13–19 years was common, with up to nine in 10 students having engaged in sexual intercourse by the study period [48]. We recommend public health messages designed for the young population to increase their knowledge and encourage them to adopt prevention approaches as a means to control cervical cancer and its devastating consequences on the individual woman and the population. Health education interventions especially at the individual level have been shown to be effective in increasing cervical cancer knowledge and promoting cervical cancer screening uptake [54–56]. In general, training interventions have been shown to effectively increase cancer knowledge and screening uptake. In Tanzania, pre-test scores on cancer knowledge was low among participants that underwent a 1-day cancer symposium. After training, knowledge scores increased with a significant difference between mean scores pre- and post-training [57]. In Ethiopia, the demand for cervical cancer screening (willingness to screen and having a plan to screen) significantly increased following a 3-days training of 674 women (340 intervention, and 344 control) [58]. In Malaysia, an interventional study showed that providing education talk to women leads to significant improvement in knowledge of cervical cancer and Pap smear, as well as attitude towards and actual uptake of Pap smear tests [56]. Since the school girls depend on friends or peers for information about cervical cancer and perhaps other health conditions, health education training interventions using peer education might be helpful in the prevention and control of cervical cancer in this study population.

The low awareness of prevention approaches in this study may relate to the fact that majority of the girls did not perceive themselves to be at risk to cervical cancer. It is human nature to not worry and not prepare to mitigate risk and or take preventive actions regarding what one does not perceive as a threat. Perceptions of risk, knowledge of the nature and perceived seriousness of the risk as well as experiences, values and cultural beliefs influence the decision to infer self-risk and preparedness to take preventive actions [59,60]. People who perceive themselves at risk seek to know about and undertake prevention measures. In a community setting among 416 women in western Uganda, women with high self-perceived risk for cervical cancer had higher intention to undertake cervical cancer screening [61]. A systematic review and meta-analysis of studies in sub Saharan Africa showed that high perceived susceptibility to cervical cancer increased the odds of cervical cancer screening uptake [62]. Similarly, low self-perceived risk for HIV has been associated with low uptake of pre-exposure prophylaxis [63]. Therefore, increasing knowledge of risk factors and people at risk of cervical cancer among school girls will likely help them to objectively assess their personal risk and influence their uptake of cervical cancer preventive approaches.

School based cervical cancer education has been shown to increase knowledge and improve attitudes and intentions to undertake HPV vaccination among 953 Chinese adolescents [64]. Similarly, providing health education to health sciences teachers in high schools in Japan significantly increased their intentions to recommend HPV vaccinations to adolescent girls in their schools [65]. There is evidence that school-based peer education and learning especially on health matters have effectiveness in learning outcomes including an increase in knowledge and change of attitudes [66,67]. Educational interventions implemented at schools do not only increase knowledge about cancers but also motivates the students who participate to act as health promoters in their families and social contacts [68]. School health educations on cervical cancer and other cancers ought to include the teachers as well as the students to achieve maximum gain for the resources invested. Such educational programs with students have multiplier effects and increase value for money as well as save the lives of the other people who learn about cervical cancer from the trained students.

The finding of the low prevalence of perceived causes of cervical cancer in this study is encouraging; this finding is contrary to an earlier study in the same region that showed older men and women believed that rough sex, having sex with a polygamous man, and sexual intercourse during menstrual periods caused cervical cancer [69]. The prevalence of the perceived causes of cervical cancer seem to increase with age as women get exposed to such knowledge or as they reason through based on unsubstantiated observations. Therefore, it is critical that tailored public health messages to discount these misperceptions be targeted to the girls at schools so they do not eventually learn and perpetuate such beliefs. Such an educational intervention proved effective in Ghana where a study that included female aged 10–19 years showed that educating women on key cervical cancer aspects including cervical cancer risk factors and symptoms, and cervical cancer susceptibility significantly improved knowledge and perceived benefits of cervical screening [70]. Similarly, a systematic review that included 19 studies conducted between 2005 and 2020 in Africa, and which assessed the impact of health education on cervical cancer awareness, knowledge, and screening and or vaccination uptake showed that educational interventions are overall effective. The authors of the review study advised that educational interventions maybe more effective when there is proper understanding and efforts to reduce on barriers to screening and vaccination uptakes [71]. Another review study that included 17 studies from all over the world conducted between 2005 and 2017 showed that educational interventions are effective in increasing cervical screening uptake [55]. We therefore recommend that targeted educational interventions to reduce the incidence and mortality from invasive cervical cancer among students and community members ought to aim at not only increasing knowledge on risk factors and symptoms, and uptake of prevention approaches but also reducing misperceptions and other barriers to cervical cancer prevention uptake and prompt health seeking for symptoms.

## Strengths and limitations

The study included all the secondary schools in the district and therefore findings are generalizable to the population in the district, and perhaps to the region. The findings may as well be generalizable to other populations with similar characteristics (e.g. post war, rural situations, culture, poverty status) as the study district. Second, this study is unique in that it has demonstrated that parents of school children can actually be involved in research studies where their children are prospective participants when class teachers who they know reach out to them to seek for their consents.

This study had some limitations; it is a cross-sectional study from which causality cannot be attributed to the observed associations. Second, although the tool for data collection was pre-tested and adjusted accordingly, the content validity and reliability evaluations were not conducted. Third, generalization of findings from this study ought to put into considerations the unique circumstance of northern Uganda as a post conflict region.

## Conclusions

Adolescent school girls have low knowledge of cervical cancer risk factors and measures to prevent the disease. Low self-perceived risks for cervical cancer in the face of early onset of penetrative vaginal sexual intercourse and low uptake of HPV vaccination ought to be addressed to reduce the incidence and advanced stage cervical cancer at diagnosis in the region.

## Future research implications and practical implications

Future interventional studies are needed to identify effective approaches to increase awareness and promote cervical cancer prevention uptake including HPV vaccination and cervical screening in this region. Findings from this study add evidence for the need of school health programs. Secondary schools ought to establish school health programs and regularly provide health education on topical matters including cervical and breast cancers as well as other non-communicable diseases including hypertension and heart diseases.

## Supplementary Material

Supplementary_Material_1_Questionnaire.pdf

## Data Availability

The data that support the findings of this study are available from the corresponding author (ADM), upon reasonable request.
