# Supplementary material for: Awareness of cervical cancer risk factors and preventive approaches, and perceived causes of cervical cancer among secondary school girls: a cross-sectional study in Northern Uganda
Source: Ann Med. 2024 Jul 8;56(1):2374860. doi: 10.1080/07853890.2024.2374860 (PMC11232641; doi:10.1080/07853890.2024.2374860)
Supplement: Supplementary_Material_1_Questionnaire.pdf [file IANN_A_2374860_SM3777.pdf]

Structured questionnaire

Awareness of cervical risk factors and preventive approaches, and sources of information and perceived causes of cervical cancer among secondary school girls: A cross-sectional study in post-conflict northern Uganda

1. Serial number: …………………………….
2. Study number (linked with participant's identifier): ………………………………..

A. Socio-demographic characteristics of participants

3. Sub-county: ………………………..
4. Parish: ……………………………...
5. Village: …………………………….
6. School name: ………………………………… Private [ ] Government [ ]
7. Age: ……………………………….. (Years).
8. Class: ……………………………….
9. Religion

1 [ ] Roman Catholic
2 [ ] Protestant/ Anglican
3 [ ] Muslim
4 [ ] Pentecostal/ Born Again
5 [ ] Traditional religion/ Ancestral Worshipers
5 [ ] Others (specify): ………………………….

B. School factors and awareness of cervical cancer

10. Presence of a school dispensary/ nurse
    1 [ ] Present
    2 [ ] Absent

11. Availability of School health programs
    1 [ ] Available
    2 [ ] Not available

12. Proximity of school to a health facility (health center/ hospital)
    1 [ ] less than 5 km
    2 [ ] more than 5 km

C. Parental factors associated with awareness of cervical cancer

13. Age of parents
    1[ ] Father: ………….. years
    2[ ] Mother: ………… years

14. Marital status of parents
    1 [ ] Married
    2 [ ] Divorced
    3 [ ] other (specify) ……………………………………………………………….

15. Level of education of parents (Tick appropriately)

**Education attainment/level**	**Mother**	**Father**
1- No formal education		
2- Primary 1 - 5		
3- Primary 6 – 7		
4- Primary 7 leaver		
5- Senior 1 – 4		
6- Senior 5 – 6		
7- Senior 6 leaver		
8- Post high school training institution		
9- University Education		
10- Don't know		

16. Occupation of parents

**Occupation**	**Mother**	**Father**
1 – Employed formally		
2 - Petty trader		
3 – Peasant farmer		
4 – Other (Please specify):<br>……………………………………………………<br>……………………………………………………<br>……………………………………….		

D. Water source factors and cervical cancer awareness

17. Distance from residence
    1 [ ] less than 1 km
    2 [ ] more than 1 km

18. Type of water source
    1 [ ] Well/ spring
    2 [ ] Tape water
    3 [ ] Borehole
    4 [ ] Other (specify): …………………………………………………………………..

E. Communal activities associated with awareness

19. Activities you participate in at home:
    1 [ ] Digging/ wedding/ harvesting
    2 [ ] Protecting water sources
    3 [ ] Digging roads
    4 [ ] Other (specify): ……………………………………………………………….

F. Health care related factors and cervical cancer awareness

20. Distance of nearest health facility from place of residence
    1 [ ] More than 5 km
    2 [ ] Less than 5 km

21. Have you ever heard of a vaccine (HPV) that can be given to young girls who have never had sex to prevent them from getting cervical cancer?
    1 [ ] Yes
    2 [ ] No

22. What is your HPV vaccination status?
    1 [ ] Vaccinated
    2 [ ] Not vaccinated

G. Cervical cancer knowledge and sources/ channels of information

23. Have you ever heard of cancer of the cervix?
    1 [ ] Yes
    2 [ ] No

24. If yes, from where or from whom did you FIRST hear of it? (tick all that apply)
    1 [ ] From neighbors
    2 [ ] Through television
    3 [ ] Through the radio
    4 [ ] Through the radio

5 [ ] From the newspapers
6 [ ] Through health education/ health units/ health workers
7 [ ] From school
8 [ ] From friends
9 [ ] From family members
10 [ ] From churches/ mosques
11 [ ] Other (specify) …………………….

25. Cancer of the cervix is a sexually transmitted disease?
    1 [ ] Yes

    2 [ ] No

    3 [ ] Do not know

G1: Perceived causes/ risk factors for cervical cancer

26. The following are most times the causes of cervical cancer in a woman; answer true (T) or false (F). [First let the participant enumerate her causes and tick in 3rd column. Then read out the remaining causes and indicate T or F in the 4th and 5th columns as she responds. (This captures both knowledge and misconceptions)].

**S/N**	**Perceived cause/ risk factor for cervical cancer**	**Answered after reading out/prompted**		
		**True (T)**	**False (F)**	**Not sure/Don't know**
1	Starting to have sex early (Before 15 years of age)			
2	Having sex with many different boys/men within same period of time			
3	Having sex with a man who is polygamous (the man has sex with many other women)			
4	Having sex with a boyfriend/husband of another girl/woman			
5	Having sex with a boy/man who does not believe in God			
6	Being cursed by the elders			
7	Because of annoying the spirit of the dead			
8	Low economic status or being poor			
9	Getting infected with some germ/ virus (Human Papillomavirus) from a boy/man			

10	Having sex with a boy/man who has rough sexual intercourse			
11	Having sex before marriage			
12	Using the family planning pills and injections, or coil			
13	Smoking cigarettes			
14	Bearing many children			
15	It is contagious; you get it when you get near to a person with it			
16	It is inheritable; you get it if your mother or aunt had it			
17	Not washing the private part well especially after sex			
18	Having sex with a boy/man when a girl/woman is in her menstrual periods			
19	Others stated by participants: …………………………………………………..			

27. Do you have a boyfriend?

1 [ ] Yes
2 [ ] No
3 [ ] Prefer not to tell

28. If yes, how many do you have currently?

1 [ ] Only one
2 [ ] More than one
3 [ ] Prefer not to tell

29. Have you ever had sexual intercourse?

1 [ ] Yes
2 [ ] No
4 [ ] Prefer not to tell

30. If yes, how old were you at your first sexual debut? …………… Years

31. Have you had sexual intercourse with more than one man?

1 [ ] Yes
2 [ ] No
3 [ ] Prefer not to tell

32. Do you perceive yourself to be at risk of cervical cancer?
    1 [ ] Yes
    2 [ ] No
    3 [ ] Don't know

Prevention of cervical cancer

33. Can cancer of the cervix be prevented?
    1 [ ] Yes
    2 [ ] No
    3 [ ] Do not know

34. If yes, how may it be prevented?

    1 [ ] Giving vaccination to young girls before sexual intercourse

    2 [ ] Getting frequent vaginal examination at health centers/ hospitals to detect it early

    3 [ ] Frequent washing of the vagina (douching), at least 2 times a day

    4 [ ] Abstinence from sex

    5 [ ] Being faithful to one sexual partner

    6 [ ] Other (Please specify): ……………………………………………………………….

Name of Interviewer: ……………………………

Signature: ………………………………………..

Date of Interview: ……………………………….

**************** Thank you for participating****************
